# TLR4 interaction with LPS in glioma CD133+ cancer stem cells induces cell proliferation, resistance to chemotherapy and evasion from cytotoxic T lymphocyte-induced cytolysis

**DOI:** 10.18632/oncotarget.18586

**Published:** 2017-06-21

**Authors:** Fengyuan Che, Jiawei Yin, Yanchun Quan, Xiaoli Xie, Xueyuan Heng, Yifeng Du, Lijuan Wang

**Affiliations:** ^1^ Department of Neurology, Shandong Provincial Hospital, Shandong University, Jinan, Shandong Province, China; ^2^ Central Laboratory, Linyi People's Hospital, Shandong University, Linyi, Shandong Province, China; ^3^ Department of Neurology, Linyi People's Hospital, Shandong University, Linyi, Shandong Province, China; ^4^ Department of Neurosurgery, Linyi People's Hospital, Shandong University, Linyi, Shandong Province, China; ^5^ Department of Hematology, Linyi People's Hospital, Shandong University, Linyi, Shandong Province, China

**Keywords:** glioma cancer stem cells, CD133-positive, toll-like receptor 4, cytotoxic T lymphocyte, immune evasion

## Abstract

Despite advances in treatment modalities, 5-year survival among glioma patients remains poor. Glioma cancer stem cells (CSCs) exhibit high tumorigenic activity and are associated with resistance to treatment and tumor recurrence. Because overexpression of toll-like receptor 4 (TLR4) correlated with cancer development, we investigated LPS-induced TLR4 signaling in glioma CD133-positive (CD133+) CSCs. The proliferation of CD133+ CSCs isolated from CSCs derived from the U251 and SF295 glioma cell lines and from human glioma samples was upregulated on a time- and concentration-dependent basis by LPS stimulation, with increases in *CD133*, *NANOG*, and *NESTIN* mRNA and protein levels. Also elevated was cytokine expression, which was coupled to phosphorylation of mitogen-activated protein kinase, and activation of cyclins and cyclin-dependent kinase complexes. *TLR4* knockdown reduced LPS-induced CD133+ CSC proliferation, whereas Adriamycin-induced CD133+ CSC apoptosis was moderately inhibited by treatment with LPS, implying a protective effect of LPS. The capacity of glioma CD133+ CSC-reactive cytotoxic T lymphocyte to selectively kill CD133+ CSCs was reduced by LPS, and this effect was not apparent after *TLR4* knockdown in CD133+ CSCs. These data suggest TLR4 signaling is a factor in CD133+ CSC immune evasion, and thus disruption of TLR4 signaling is a potential therapeutic strategy in glioma.

## INTRODUCTION

Gliomas account for approximately 80% of all malignant primary central nervous system tumors [1, 2]. Stage I and II gliomas are lower-risk tumors and have a better prognosis, whereas stage III and IV gliomas, including anaplastic astrocytomas and glioblastomas, are high-grade malignant tumors [3, 4]. Although progress has been made in treatment modalities such as surgery, chemotherapy, and radiotherapy, recurrence after standard therapies is inevitable, and the median survival of patients with high-grade gliomas is no more than 14 months [5, 6]. The 5-year survival rate of patients with glioblastoma is less than 3% [4]. Thus, finding susceptible cells and molecules on which to apply new therapeutic options has been the focus of diagnosis and treatment of gliomas.

Studies confirm the presence of cancer stem cells (CSCs) in various cancer tissues [7, 8]. CSCs are undifferentiated cells within tumors that possess high tumorigenic activity and the capacity to self-renew and undergo multilineage differentiation [9–11]. CSCs possess biological characteristics such as rapid repair of damaged DNA [12, 13], metabolic reprogramming [14], adaption to hyperinflammatory microenvironments [10], and resistance to oxidative stress and anticancer drugs [10, 15]. Evidence indicates that malignant glioma CSCs induce cancer and promote cancer development [16, 17]. Moreover, glioma CSCs promote cancer resistance to treatment and recurrence, leading to high mortality [18]. Although opinions differ, most reports define CD133-positivity (CD133+) as a marker of glioma CSCs [19–21].

Evidence indicates that inflammation is a factor in the initiation, proliferation, progression, and dissemination of certain cancers [22–26]. Inflammation is induced by direct reaction with pathogens [27]. Toll-like receptors (TLRs) are type I transmembrane molecules that react with foreign pathogens such as bacteria and viruses, and trigger immune responses [28, 29]. TLRs are correlated with development and progression of various tumors [30]. Toll-like receptor 4 (TLR4) is expressed on the cell surface and reacts with the membrane components of foreign pathogens [29, 31]. TLR4 reacts with lipopolysaccharide (LPS) in the cell wall of Gram-negative bacteria [32]. TLR4 is expressed in various cancers and functions in cancer biology [33, 34]. In colorectal cancer (CRC), increased TLR4 levels are correlated with cancer stage, histological grade, metastasis, progression, and prognosis of CRC patients [34–36]. LPS enhances the expression of VEGF-C, which promotes cell motility and metastasis by triggering TLR4 signaling, and *TLR4*-deficient mice are protected from CRC oncogenesis [34, 37]. Activation of the TLR4 signaling pathway results in cancer cell proliferation and angiogenesis in pancreatic cancer [38, 39]. LPS-triggered TLR4 signaling enhances cancer cell proliferation and leads to drug resistance in hepatoblastoma cells [40]. LPS promotes the stemness of CD133+ CSCs in hepatoma [32]. However, little is known about the function of LPS-induced TLR4 signaling in glioma CD133+ CSCs. Therefore, we investigated the function of TLR4 in glioma CD133+ CSCs. We found that LPS promotes the proliferation and drug resistance of glioma CD133+ CSCs and leads to immune evasion of CD133+ CSCs from cytotoxic T lymphocyte-induced cytolysis.

## RESULTS

### Identification of glioma CSCs

**Figure 1:**
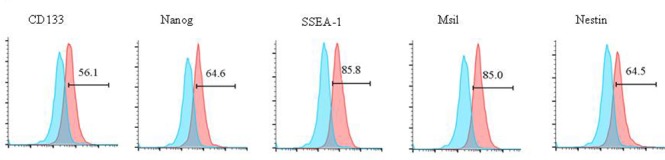
Identification of glioma CSCs via flow cytometry Surface proteins of CD133, Nanog, SSEA-1, Msil, and Nestin are detected by flow cytometry in CSCs generated from one glioma patient.

We generated CSCs from glioma cell lines or patient samples and identified CSCs by flow cytometry. The expression of CSC markers, including CD133, Nanog, SSEA-1, Msil and Nestin in glioma CSCs generated from one glioma patient are shown in Figure 1.

### TLR4 is expressed in human glioma CD133+ CSCs and human glioma tissues

**Figure 2:**
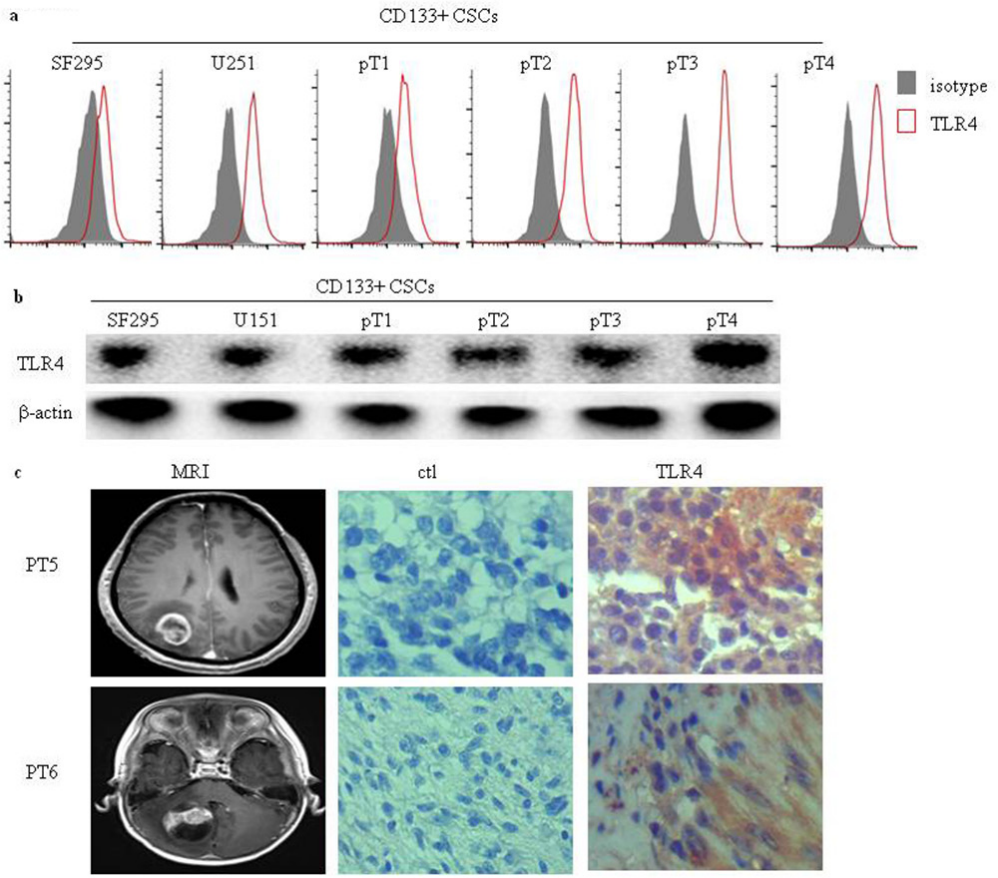
TLR4 expression in human glioma CD133+ CSCs and glioma tissues Surface **(a)** and total **(b)** TLR4 protein expression in six human glioma CD133+ CSCs isolated from CSCs derived from SF295 and U251 and four fresh human surgical glioma tissue samples (pT1 to pT4) was detected by flow cytometry and Western blot analysis. **(c)** Representative MRI images and immunochemistry staining of TLR4 expression in two glioma patients. An isotype control antibody was used for negative control staining. Original magnification ×400.

We investigated the expression of TLR4 in human glioma CD133+ CSCs isolated from six CSCs derived from two glioma cell lines, SF295 and U251, and four fresh human surgical glioma tissues, patient (pT) 1 to pT4. Flow cytometry (Figure 2a) and Western blot analysis (Figure 2b) demonstrated that TLR4 protein was expressed in six human CD133+ glioma CSCs. Magnetic resonance imaging (MRI) was performed to identify intracranial gliomas of two patients (Figure 2c). TLR4 expression was analyzed in two glioma patients by immunohistochemistry (IHC). We identified expression of TLR4 protein in glioma tissues (Figure 2c).

### LPS stimulation induces proliferation of CD133+ CSCs and protects CD133+ CSCs from Adriamycin-induced apoptosis

**Figure 3:**
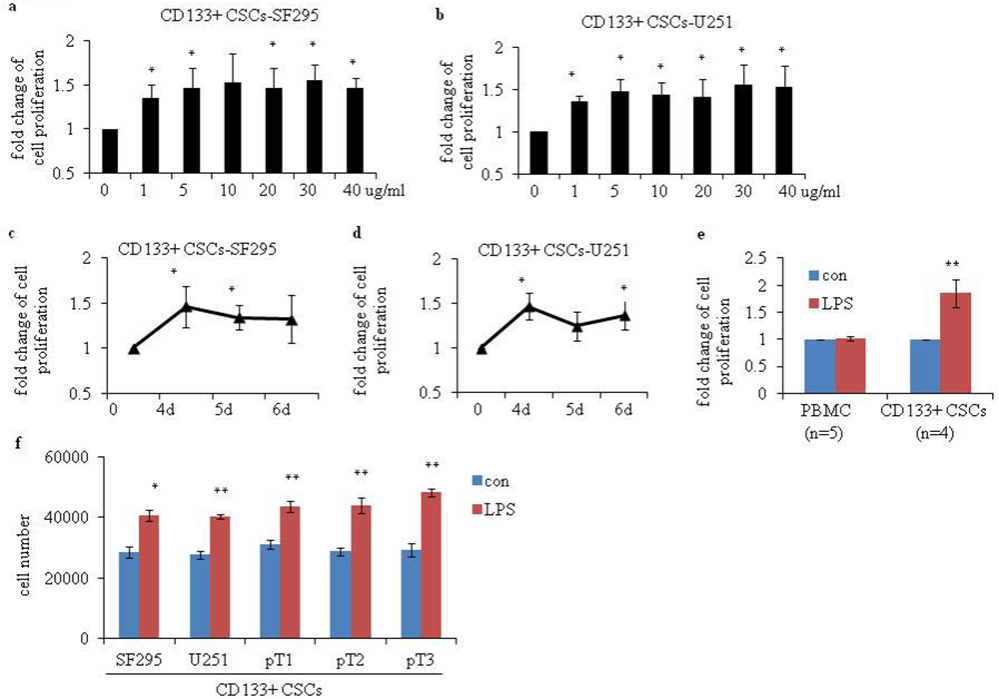
Proliferation of human glioma CD133+ CSCs after stimulation with LPS CCK-8 assays were performed to determine the proliferation of CD133+ CSCs isolated from CSCs derived from **(a)** SF295 (CD133+ CSCs-SF295) and **(b)** U251 (CD133+ CSCs-U251) after stimulation with 0 to 40 μg/mL LPS for 4 days. Proliferation of **(c)** CD133+ CSCs-SF295 and **(d)** CD133+ CSCs-U251 after stimulation with 5 μg/mL LPS for 4, 5, and 6 days. **(e)** Proliferation of five PBMC samples (isolated from five healthy donors) and four CD133+ CSCs derived from four fresh human surgical glioma tissues (pT1 to pT4) after stimulation with 1 μg/mL LPS for 4 days. **(f)** Cell number was counted to determine the proliferation of CD133+ CSCs isolated from CSCs derived from SF295, U251, pT1, pT2 and pT3 after stimulation with 1 μg/mL LPS for 6 days. *P < 0.05, **P < 0.01, compared with the control (con) cells.

**Figure 4:**
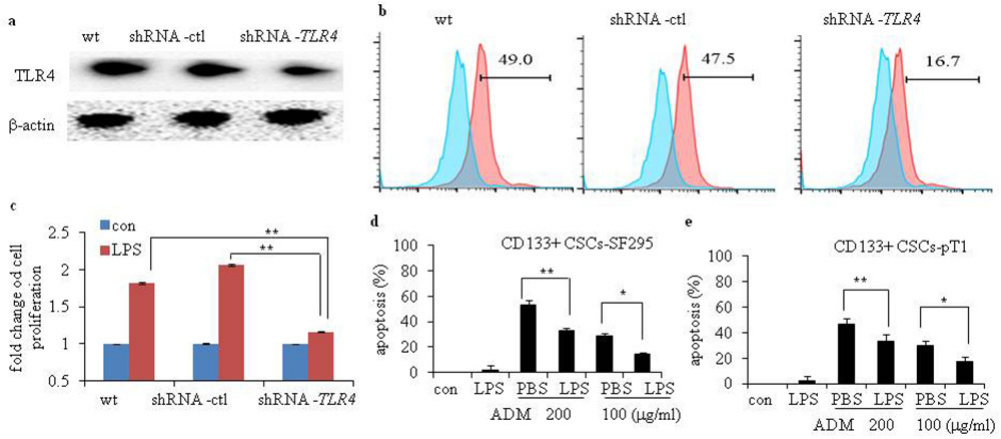
Efficiency of TLR4 knockdown and chemotherapy-induced apoptosis of human glioma CD133+ CSCs after stimulation with LPS Efficiency of TLR4 knockdown in wild-type (wt) CD133+ CSCs and cells transfected with control shRNA (shRNA-ctl) or *TLR4*-specific (shRNA-*TLR4*) lentiviral particles were evaluated by Western blot analysis **(a)** and flow cytometry **(b)**. **(c)** Proliferation of wt, shRNA-ctl, and shRNA-*TLR4* CD133+ CSCs. **(d)** LPS protected CD133+ CSCs-SF295 from ADM-induced apoptosis. **(e)** LPS protected CD133+ CSCs-pT1 from ADM -induced apoptosis. *P < 0.05, **P < 0.01, compared with the control (con) cells.

To study the activity of TLR4 in human glioma CD133+ CSCs, we incubated CD133+ CSCs isolated from CSCs derived from SF295 and U251 glioma cell lines with LPS at different concentrations and time points. The proliferation of CD133+ CSCs was upregulated on a dose-dependent (Figure 3a and 3b; *P* < 0.05, compared to CD133+ CSCs without LPS stimulation) and time-dependent (Figure 3c and 3d; *P* < 0.05, compared to CD133+ CSCs without LPS stimulation) basis. After stimulation with LPS, CD133+ CSCs isolated from four CSC samples derived from four fresh human surgical glioma tissues, pT1 to pT4, also demonstrated enhanced proliferation (Figure 3e; *P* < 0.01, compared to CD133+ CSCs without LPS stimulation), whereas PBMCs did not (Figure 3e). The number of CD133+ CSCs increased after LPS stimulation compared with cells without LPS stimulation (Figure 3f; *P* < 0.05). We used Pam3C to activate TLR2 signaling and found that TLR2 activation did not promote the proliferation of CD133+ CSCs (data not shown). To verify the function of TLR4 in the proliferation of CD133+ CSCs, TLR4 expression in CD133+ CSCs was knocked down by use of *TLR4*-specific short-hairpin RNA (shRNA) lentiviral particles. In the present study, CD133+ CSCs were classified as wild-type (wt), control-shRNA transfected (shRNA-ctl), and *TLR4*-shRNA transfected (shRNA-*TLR4*) cells. Western blot (Figure 4a) and flow cytometry analysis (Figure 4b) revealed that shRNA-*TLR4* downregulated the expression of TLR4. Compared with wt or shRNA-ctl CD133+ CSCs, *TLR4* knockdown resulted in a reduced response of CD133+ CSCs to LPS exposure (Figure 4c; *P* < 0.01).

We next examined the function of LPS on chemotherapy-induced apoptosis of CD133+ CSCs. We determined that Adriamycin (ADM, 200 μg/mL or 100 μg/mL) is an effective chemotherapy drug for CD133+ CSCs. Preincubation of CD133+ CSCs derived from SF295 and one fresh human surgical glioma tissue sample, pT1, with LPS partially inhibited ADM-induced apoptosis (Figure 4d and 4e; *P* < 0.01).

### LPS exposure promoted the expression of gene markers in CD133+ CSCs

**Figure 5:**
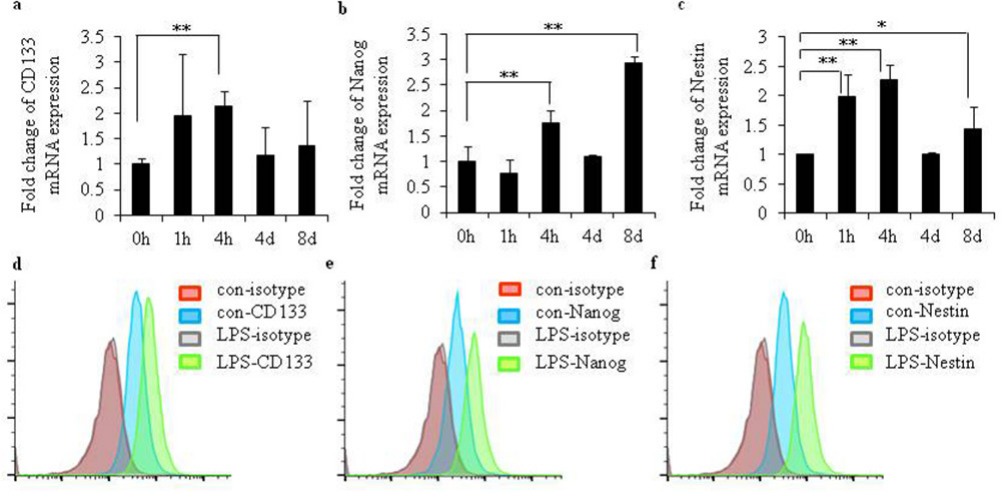
LPS increased expression levels of gene markers in glioma CD133+ CSCs mRNA levels of **(a)** CD133, **(b)** Nanog, and **(c)** Nestin after stimulation with LPS (25 μg/mL) for 0 hours, 1 hour, 4 hours, 4 days, and 8 days in glioma CD133+CSCs isolated from CSCs generated from U251. Surface protein expression of **(d)** CD133, **(e)** Nanog, and **(f)** Nestin after stimulation with LPS (25 μg/mL) for 24 hours in glioma CD133+CSCs isolated from CSCs generated from U251. *P < 0.05, **P < 0.01, compared with the control groups.

To investigate the function of LPS in the regulatory expression of surface markers in CD133+ CSCs isolated from CSCs generated from U251, the mRNA expression of CD133, Nanog, SSEA-1, Msil, and Nestin were examined. After LPS exposure, the mRNA levels of CD133 (Figure 5a; *P* < 0.01), Nanog (Figure 5b; *P* < 0.01), and Nestin (Figure 5c; *P* < 0.01) increased, compared to CD133+ CSCs without LPS stimulation. We detected the surface protein expression of CD133, Nanog, SSEA-1, Msil, and Nestin by flow cytometry. The surface expression of CD133 (Figure 5d), Nanog (Figure 5e), and Nestin (Figure 5f) increased after LPS stimulation. No changes of mRNA and protein levels of SSEA-1 and Msil were observed (data not shown).

### LPS enhances the production of cytokines in glioma CD133+ CSCs

**Figure 6:**
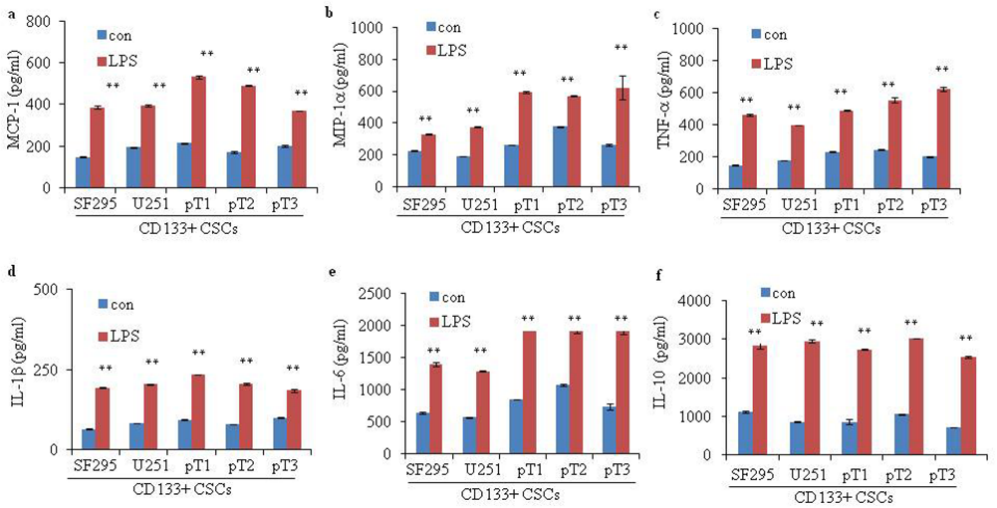
Cytokine secretion of glioma CD133+ CSCs upon LPS stimulation Secretion of **(a)** MCP-1, **(b)** MIP-1α, **(c)** TNF-α, **(d)** IL-1β, **(e)** IL-6, and **(f)** IL-10 in CD133+ CSCs derived from two glioma cell lines, SF295 and U251, and three fresh human surgical glioma tissue samples, pT1 to pT3, without or with pretreatment with LPS (1 μg/mL) for 4 days. **P < 0.01, compared with the control (con) groups.

We examined LPS-induced secretion of cytokines in glioma CD133+ CSCs isolated from CSCs derived from SF295 and U251 and three fresh human surgical glioma tissue samples, pT1 to pT3. We incubated CD133+ CSCs with LPS for 96 hours and collected the supernatants for enzyme-linked immunosorbent assay (ELISA). Compared to CD133+ CSCs without LPS stimulation, levels of monocyte chemotactic protein 1 (MCP-1) (Figure 6a; *P* < 0.01), macrophage inflammatory protein-1 alpha (MIP-1α) (Figure 6b; *P* < 0.01), tumor necrosis factor-alpha (TNF-α) (Figure 6c; *P* < 0.01), interleukin-1 beta (IL-1β) (Figure 6d; *P* < 0.01), interleukin-6 (IL-6) (Figure 6e; *P* < 0.01), and interleukin-10 (IL-10) (Figure 6f; *P* < 0.01) were elevated after LPS stimulation.

### Proteins and signaling pathways associated with TLR4 activation in glioma CD133+ CSCs

**Figure 7:**
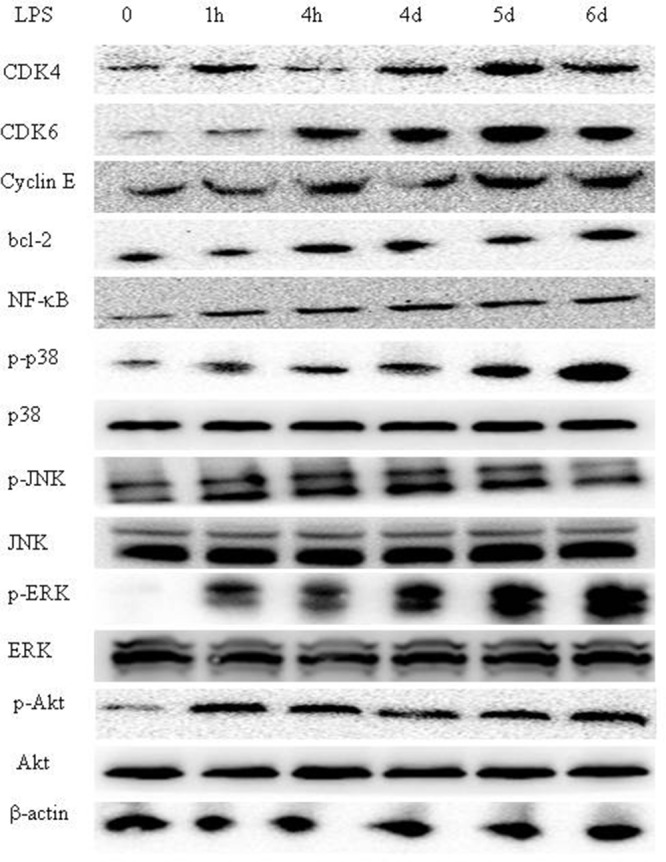
LPS triggers the TLR4 signaling pathway in glioma CD133+ CSCs Expression of CDK4, CDK6, cyclin E, bcl-2, NF-κB, phosphorylated p38, p38, phosphorylated JNK, JNK, phosphorylated ERK, ERK, phosphorylated Akt, Akt, and β-actin in glioma CD133+ CSCs isolated from CSCs generated from SF295 after stimulation with LPS (1 μg/mL) at different time points (0 hours to 6 days).

LPS stimulation-activated signaling pathways in glioma CD133+ CSCs isolated from CSCs generated from SF295 were further investigated. First, we found that LPS activated cyclin and cyclin-dependent kinase (CDK) complexes, including CDK4, CDK6, and cyclin E in CD133+ CSCs (Figure 7). Second, we analyzed the expression of apoptosis-related molecules such as bcl-2, survivin, and BAX in CD133+ CSCs. The protein expression of bcl-2 increased (Figure 7), whereas there was no obvious change of survivin and BAX (data not shown) after LPS stimulation. Third, we demonstrated that LPS exposure increased the levels of nuclear transcription factor-κB (NF-κB), phosphorylated p38 (p-p38), phosphorylated c-Jun N-terminal kinase (p-JNK), phosphorylated extracellular signal-regulated kinase (p-ERK), and phosphorylated protein kinase B (p-Akt) in CD133+ CSCs (Figure 7). The total protein of p38, JNK, ERK, and Akt did not increase after LPS stimulation (Figure 7).

### TLR4 signaling facilitates immune evasion of glioma CD133+ CSCs

**Figure 8:**
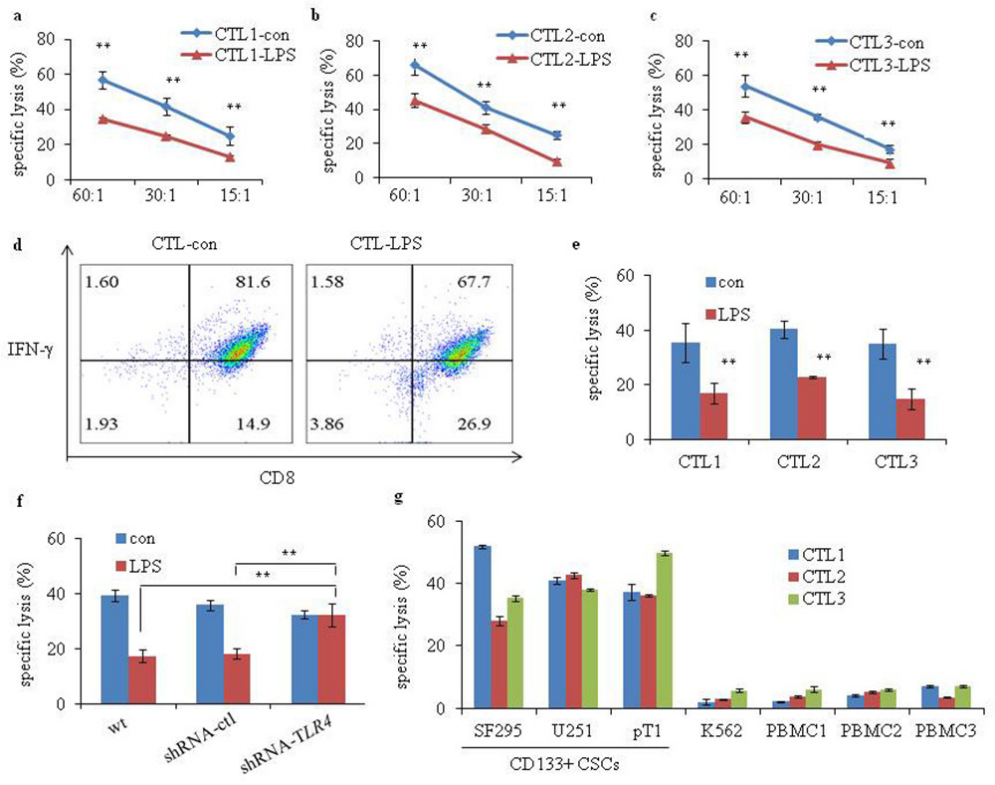
Cytotoxicity of glioma CD133+ CSC-reactive CTL Cytotoxicity of **(a)** CTL1-con and CTL1-LPS (generated without or with irradiated LPS-stimulated glioma CD133+ CSCs isolated from CSCs generated from SF295), **(b)** CTL2-con and CTL2-LPS (generated without or with irradiated LPS-stimulated glioma CD133+ CSCs isolated from CSCs generated from U251) and **(c)** CTL3-con and CTL3-LPS (generated without or with irradiated LPS-stimulated glioma CD133+ CSCs isolated from CSCs generated from one fresh human surgical glioma tissue, pT1) against target cells. The ratios of effector cells: target cells were 60:1, 30:1, and 15:1, respectively. A ratio of 30:1 was used for the following function assay. **(d)** Flow cytometry analysis showing the percentages of IFN-γ-expressing CD8+ T cells in the CTL-con and CTL-LPS. **(e)** CTL1 to CTL3 against specific CD133+ CSCs pretreated without or with LPS (1 μg/mL) for 4 days. **(f)** CTL against CD133+ CSCs (wt, shRNA-ctl, and shRNA-*TLR4*) pretreated without or with LPS (1 μg/mL) for 4 days. **(g)** Cytotoxicity of CTL1 to CTL3 against target cells, including CD133+ CSCs isolated from CSCs derived from U251, SF295 and pT1, K562, and PBMC (PBMC1-PBMC3). K562 was used as a control for NK-cell activity. **P < 0.01, compared with the control (con) groups.

We investigated the function of TLR4 signaling in cytotoxic T lymphocyte (CTL)-induced cytolysis to glioma CD133+ CSCs. CD133+ CSC-reactive CTL1 to CTL3 were established via co-culture of CD3+ T cells with three types of irradiated CD133+ CSCs derived from SF295 and U251 and one fresh human surgical glioma tissue sample, pT1, that were pretreated with or without LPS. The sensitivity of CTL generated against target LPS-pretreated CD133+ CSCs (CTL-LPS) was significantly reduced compared with CTL generated against target control CD133+ CSCs (CTL-con) (Figure 8a, 8b and 8c; *P* < 0.01). Flow cytometry analysis showed the proportion of CD8+ T cells expressing interferon- γ (IFN-γ) was lower in the CTL-LPS than in the CTL-con (Figure 8d). Moreover, pretreatment of CD133+ CSCs cells with LPS before cytotoxic assays inhibited the killing capacity of CTL compared with pretreatment with medium only (Figure 8e; *P* < 0.01). However, when pretreated with LPS, CD133+ CSCs transfected with shRNA-*TLR4* were more sensitive than control or wt cells to CTL-induced killing (Figure 8f; *P* < 0.01). These results suggest that TLR4 signaling is involved in immune evasion by glioma CD133+ CSCs. We also determined whether the CTL kill normal cells and K562 cells. Purified normal peripheral blood mononuclear cells (PBMC) from glioma patients and K562 cells were used in the test and it demonstrated that less killing occurred in normal PBMC or K562 cells, whereas CTL kill glioma CD133+ CSCs (Figure 8g).

### Glioma CD133+ CSC-reactive CTL is memory effector T cell

**Figure 9:**
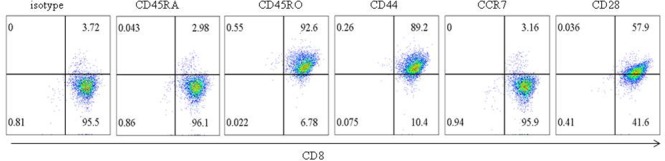
Phenotype of glioma CD133+ CSC-reactive CTL The surface expression of CD45RA, CD45RO, CD44, CCR7, and CD28 in glioma CD133+ CSC-reactive CTL was examined by flow cytometry.

We detected the surface expression of CD28, CD45RA, CD45RO, CCR7, and CD44 in CTL and found CD28, CD45RO, and CD44 were expressed in glioma CD133+ CSC-reactive CTL, whereas CD45RA and CCR7 were rarely expressed (Figure 9). Thus, memory effector T cells function in LPS-induced immune evasion.

## DISCUSSION

Previous studies reported TLR4 to be absent on glioblastoma cells, and LPS-induced antitumoral effects might depend on microglia and inflammatory cells [41]. No TLR4 expression was observed in astrocytes and oligodendroglial cells [42]. However, another study demonstrates that TLR4 is expressed on human astrocytes and glioma cell lines GL261, U373MG, U118, and U87 [43–46]. Immunohistochemical detection revealed that TLR4 protein is overexpressed in tissues from glioblastoma patients [47]. LPS-activated TLR4 signaling promotes the proliferation of glioma cell lines [43, 46]. LPS also induces chemotactic migration of glioma cells [48]. Although opinions differ, it is accepted that TLR4 is expressed in glioma. However, the expression and function of TLR4 in glioma CD133+ CSCs are unknown. In the present study, we generated CSCs from glioma cell lines and patient samples. We identified CSCs by detection of surface expression of CD133, Nanog, SSEA-1, Msil, and Nestin via flow cytometry. Glioma CD133+ CSCs were isolated by use of the MACS CD133 kit for further study. We demonstrated that surface and total TLR4 protein is expressed in glioma CD133+ CSCs. IHC analysis demonstrated that TLR4 is expressed in the tissue of glioma patients. And LPS was used to investigate the function of the TLR4 signaling pathway in glioma CD133+ CSCs. LPS-activated TLR4 signaling promotes the proliferation and chemoresistance of glioma CD133+ CSCs and triggers the release of various cytokines, resulting in the resistance of glioma CD133+ CSCs to CTL-induced lysis. Knockdown of *TLR4* by shRNA in glioma CD133+ CSCs inhibited LPS-stimulated CSC proliferation and reversed the inhibitory effect of LPS-induced glioma CD133+ CSCs on the cytotoxicity of CTLs. These effects were mediated by memory effector T cells. Thus, TLR4 expression in glioma CD133+ CSCs may lead to cancer progression and immune evasion.

A previous study reported that LPS maintains the stemness of CD133+ CSCs by facilitating the expression of CD133 [32]. Because CD133, Nanog, and Nestin are considered markers of glioma CSCs [49], we examined the gene and protein levels of CD133, Nanog, and Nestin in glioma CD133+ CSCs. After LPS stimulation, the mRNA and protein levels of CD133, Nanog, and Nestin increased in glioma CD133+ CSCs. Thus, LPS helped preserve the stemness of glioma CD133+ CSCs. Whether LPS helped the colony formation, migration, and invasion of glioma CD133+ CSCs remains to be investigated.

Glioma cells produce various inflammatory mediators, such as IL-1β, IL-6, IL-8, IL-10, MCP-1, and MIP [22, 50]. Increased expression of IL-1β, IL-6, and IL-8 was also observed in glioma patient samples and was correlated with cancer invasiveness and survival of patients [50–52]. IL-1β activates signaling pathways and induces the expression of genes that promote carcinogenesis and cancer progression [22]. IL-6 signaling is essential for the proliferation, differentiation, aggressiveness, and migration of glioma cells [22, 53, 54]. IL-6 also enhances neurosphere formation capacity and promotes the stemness of glioma CSCs [55]. Elevated IL-8 expression is associated with enhanced growth, angiogenesis, and invasion of glioma [56–59]. In our present study, activation of the TLR4 signaling pathway promoted the production of IL-1β, IL-6, IL-10, MCP-1, MIP-1α, and TNF-α in glioma CD133+ CSCs. Consistent with our findings, LPS exposure induces release of IL-1β and IL-6 in U373-MG human astrocytoma cells [46]. LPS promotes the release of TNF-α and MIP-1α from monocytes [60, 61], and TNF-α induces the release of IL-8 [62, 63]. In lung cancer cells, LPS-triggered TLR4 activation enhances the secretion of IL-10 [64]. Thus, LPS-activated interaction between cytokines and glioma CD133+ CSCs might establish a complicated microenvironment that facilitates cancer progression and immune evasion.

Cell proliferation is activated by CDKs and cyclin proteins [65]. CDK4/6 and cyclin E are necessary to promote transition of cells from G1 to S phase of the cell cycle and are factors in cell adhesion [66, 67]. We found that LPS induced the expression of CDK4/6 and cyclin E in glioma CD133+ CSCs, which might promote increased proliferation of these cells. LPS also increased the protein expression of bcl-2.

LPS-triggered TLR4 activation in turn triggers the mitogen-activated protein kinase signaling pathway [68], which promotes tumor cell proliferation and facilitates inflammation [69, 70]. The phosphatidylinositol 3 kinase (PI3K)/Akt pathway is a factor in the cell cycle and apoptosis [65]. LPS stimulation increases the expression of p-p38, p-JNK, p-ERK and p-Akt in glioma CD133+ CSCs.

We generated glioma CD133+ CSC-reactive CTL by co-culturing isolated CD3+ T cells from healthy donors with different glioma CD133+ CSCs as antigen-presenting cells. We investigated CD133+ CSC-reactive CTL-induced killing of glioma CD133+ CSCs with CD8+ T cells sorted from the generated CD133+ CSC-reactive CTL. Compared with the control cells, CTL primed with LPS-stimulated CD133+ CSCs had less capacity to kill glioma CD133+ CSCs. Moreover, preincubation of glioma CD133+ CSCs with LPS inhibited CD133+ CSC-reactive CTL lysis of target CD133+ CSCs. This effect was reversed by shRNA-*TLR4* in glioma CD133+ CSCs. CD133+ CSC-reactive CTL did not kill normal PBMC or K562 cells. We proved that memory effector T cells function in LPS-induced immune evasion. A previous study reported that TLR4 ligand LPS might be a factor in cancer immune evasion. LPS-activated TLR4 signaling renders tumor cells resistant to CTL-induced killing, which might be attributed to increased expression of B7-H1, B7-H2, and CD40 and decreased expression of Fas [68]. TLR4 signaling in human mantle cell lymphoma cells inhibited T cell proliferation and CTL-induced cytolysis, and this effect was partially restored by neutralization of IL-10 and/or VEGF [71]. LPS-pretreated multiple myeloma cells suppressed the generation of CTL by inducing expression of PD-L1 [72].

Our study demonstrated the importance of TLR4-induced effects on glioma CD133+ CSCs. Further investigations are needed to elucidate TLR4-induced signaling pathways in the pathogenesis and immune evasion of glioma CD133+ CSCs. TLR4 might provide novel therapeutic approaches for patients with glioma.

## MATERIALS AND METHODS

### Cell culture and isolation of glioma CD133+ CSCs

Glioma cell line U251 and SF295 (Cell Bank of the Chinese Academy of Sciences, Shanghai, China) were cultured in Dulbecco Modified Eagle's medium (DMEM) (Hyclone,USA) supplemented with 10% fetal bovine serum (Gibco, USA) and penicillin/streptomycin (1×; Gibco, USA) at 37°C in a 5% CO_2_ incubator. The medium was changed every 2 to 3 days. Fresh glioma tissues were collected from patients undergoing craniotomy at the Department of Neurosurgery, Linyi People's Hospital (Linyi, Shandong Province, China). All patients gave written consent. To obtain CSCs from U251 or SF295 cells (CSCs-U251 or CSCs-SF295), cells were digested with collagenase D (Roche, Switzerland) and DNase I (Sigma-Aldrich, USA) when they had reached logarithmic growth phase, and then resuspended in serum-free medium (SFM) consisting of DMEM/F12 medium (Hyclone), B27 Supplement (1×; Gibco, USA), human EGF (20 ng/mL; PeproTech, USA), human FGF-basic (20 ng/mL; PeproTech, USA), leukemia inhibitory factor (10 ng/mL; PeproTech, USA), L-glutamine (2 mM; Gibco, USA), and penicillin/streptomycin (1×; Gibco, USA). SFM was renewed every 3 to 4 days and cells grew as spheres. CSCs derived from glioma patients were obtained according to the protocols described previously [49]. When cells had reached a high density at the third passage, CD133+ CSCs were isolated by use of the MACS CD133 kit (Miltenyi Biotec, Germany).

### RNA extraction, cDNA synthesis and real-time quantitative PCR

Details of RNA extraction and cDNA synthesis were described previously [71]. Real-time quantitative PCR (qPCR) was performed in triplicate for each sample on an Applied Biosystems QuantStudio 5 PCR system (Waltham, USA) with SYBR Green Real-time PCR Master Mix (TOYOBO, Osaka, Japan). Primers are listed in Table 1. The qPCR conditions were pre-denaturation at 95°C for 1 minute, followed by 40 cycles at 95°C for 15 seconds, 60°C for 15 seconds, and 72°C for 60 seconds. Data were collected and analyzed by ABI QuantStudio Design & Analysis Software Version 1.3.1. The mRNA levels of specific genes in each sample were normalized to *GAPDH* mRNA levels by use of mean values of triplicates and expressed as relative expression compared with *GAPDH*.

**Table 1:** Primer sequences for qPCR

Gene	Forward (5′→3′)	Reverse (5′→3′)
CD133	CCCGTGGATGCAGAACTTGA	CCTGAATAGGAAGACGCTGAGT
Nanog	CCCAGCTGTGTGTACTCAAT	CAGGCATCCCTGGTGGTAG
Nestin	CTGAAAAGTTCCAGCTGGCTGT	GCTGAGGGACATCTTGAGGTG
GAPDH	CGGAGTCAACGGATTTGGTCGTAT	AGCCTTCTCCATGGTGGTGAAGAC

### Flow cytometry

Antibodies against human TLR4, CD133, Nestin, SSEA-1, Msil, CD28, CD45RA, CD45RO, CCR7, CD44, IFN-γ and isotype control were purchased from eBioscience (San Diego, USA). Antibody against human Nanog was purchased from BD Biosciences (USA). Data were obtained with a flow cytometer (FACS Canto; BD Biosciences, USA).

### Immunohistochemistry

IHC was performed according to previous literature [50]. The primary antibody against TLR4 and the isotype-negative control antibody (both Abcam, UK) were used at a dilution of 1:200.

### Cell proliferation detection

CD133+ CSC proliferation was detected by use of a Cell Counting Kit-8 (CCK-8) or cell number counting. CD133+ CSCs (2 × 10^4^/200 μL per well) were cultured in 96-well culture plates (Corning Inc., Corning, USA) and treated with LPS (Sigma, USA) at different time points or concentrations. At the end of each experiment, CCK-8 was added to the culture system for 3 hours and detected, by use of a microplate reader (Bio-Rad, USA).

### Western blot

Details of the Western blot analysis were described previously [43]. Phosphorylated p38, phosphorylated JNK, phosphorylated ERK, p38, JNK, ERK, and β-actin monoclonal antibodies were used (all Abcam, UK). Phosphorylated Akt, Akt, bcl-2, survivin, BAX, CDK4, CDK6, and cyclin E monoclonal antibodies were purchased from Cell Signaling Technology (Beverly, USA).

### ELISA

ELISA kits (all from eBioscience, USA) were used to detect the levels of IL-1β, IL-6, IL-10, MCP-1, MIP-1α, and TNF-α in cell supernatants collected from glioma CD133+ CSCs according to the manufacturer's instructions.

### *TLR4* shRNA transfection of glioma CD133+ CSCs by lentivirus

*TLR4*-specific or control shRNA lentiviral particles were obtained from Santa Cruz Biotechnology Inc. (Dallas, USA), and glioma CD133+ CSCs were transfected according to the manufacturer's protocol.

### Generation of CD133+ CSC-reactive CTL and function assays

CD3+ T cells were co-cultured with irradiated CD133+ CSCs to generate glioma CD133+ CSC-reactive CTL, which were expanded for function assays, as described previously [73]. CTL1 was generated against irradiated CD133+ CSCs isolated from CSCs derived from SF295, CTL2 was generated against irradiated CD133+ CSCs isolated from CSCs derived from U251, and CTL3 was generated against irradiated CD133+ CSCs isolated from CSCs derived from one fresh human surgical glioma tissue, pT1. CTL-con was generated against untreated glioma CD133+ CSCs and CTL-LPS were generated against glioma CD133+ CSCs stimulated with LPS. For cytotoxic assays, CD8+ T cells were sorted by use of the MACS CD8 kit (Miltenyi Biotec, Germany). A cytotoxicity assay kit (Promega Corporation, USA) was used to measure the killing capacity of CTL against target glioma CD133+ CSCs.

### Statistical analysis

Student's *t*-test was applied to compare groups and *P* values < 0.05 were considered statistically significant. Unless otherwise indicated, data are presented as the mean ± standard deviation.
